# Mendelian randomization reveals a causal link between age at menarche and reduced risk of periodontitis

**DOI:** 10.1097/MD.0000000000043700

**Published:** 2025-08-08

**Authors:** Yan Hou, Weilun Cai, Hua Xu, Jiandong Ban

**Affiliations:** a Department of Stomatology, Hebei Eye Hospital, Xingtai, China; b Department of Stomatology, Nanfang Hospital, Southern Medical University, Guangzhou, China.

**Keywords:** AAM, menarche, Mendelian randomization, periodontitis

## Abstract

The aim of this study was to assess the causal association between age at menarche (AAM) and periodontitis by use of a 2-sample 2-way Mendelian randomization (MR) study. A 2-sample bidirectional MR analysis was performed based on genome-wide association study data from European populations of AAM and chronic periodontitis, using single nucleotide polymorphisms as instrumental variables. Inverse-variance weighting, weighted median, weighted multinomial, and MR-Egger were used to assess the bidirectional causal association between AAM and chronic periodontitis. The inverse-variance weighting method showed a significant negative correlation between genetically predicted AAM and chronic periodontitis (odds ratio: 0.733; 95% confidence interval: 0.583–0.922; *P* = .0081), Cochran *Q* test showed no heterogeneity, MR-Egger showed there was no pleiotropic, leave-one-out confirmed the robustness of the results, and reverse MR analysis demonstrated no reverse causality. AAM may be negatively associated with chronic periodontitis.

## 1. Introduction

Periodontitis is one of the world’s most problematic issues of global concern impacting health with millions suffering across different populations.^[1,2]^ This form of oral disease can cause progressive destruction of the supporting structures of the teeth, resulting in inflammation of the gingivitis, tooth instability, and ultimately tooth loss.^[3]^ Even with the advances in dental care and providing knowledge of oral hygiene, periodontal diseases are still prevalent which shows the need for a better understanding of their causes and risks involved.

Smoking, improper oral hygiene, and genetic factors are widely perceived as part of these causative factors that lead to periodontal diseases.^[4,5]^ More attention has been paid to the effect of hormones, specifically to the age at menarche (AAM) in relation to oral health in more recent studies. Menarche is the age that a woman begins menstruation and symbolizes the milestones of woman reproductive capabilities and associated hormonal alterations.^[6]^ During these specific periods, sex hormones mainly estrogen and progesterone significantly alter the immune response and inflammatory response in the oral cavity.^[7]^ Moreover, these hormones probably also play prominent roles in the regulation of gingival pro-inflammatory cytokines expression and vascular reactivity to the microbiota in the gingiva. Estrogens are known to improve immune function, but at the same time they can increase the susceptibility of periodontal tissues to bacterial invasion and subsequently aggravate inflammation. Progesterone has also been found to stimulate the production of collagenase, an enzyme implicated in the breakdown of periodontal tissues. Therefore, it leads to the destruction of periodontal tissues and high susceptibility for periodontal diseases. Hormonal effects may be one of the major reasons to induce or aggravate periodontal diseases at certain sensitive periods like menarche when there are gross disturbances in the levels of hormones.

The exact association between the AAM and the incidence of periodontal diseases is complex and not particularly well defined. To establish a clearer and more causal relationship between the 2, further innovative research approaches are needed.

Mendelian randomization (MR) is a robust genetic epidemiological technique that utilizes genetic variation as an instrumental variable (IV) to explore causal relationships.^[8]^ It employs genetic markers related to the specific exposure, namely the AAM in this study, to evaluate the possible effects it may have on the risk of developing periodontal diseases. MR effectively mitigates the effects of confounding variables and lessens the biases usually seen in conventional observational studies by using the random mix of genetic variations generated during meiosis.^[9]^

In this paper, we present the results of a MR analysis investigating the association between AAM and the risk of periodontitis. By utilizing large-scale genetic data from diverse populations and combining it with epidemiological evidence, we aim to shed light on the potential role of hormonal factors in oral health and offer new insights into the prevention and management of periodontal diseases.

## 2. Materials and methods

### 2.1. Data source and IVs

**Figure 1.**
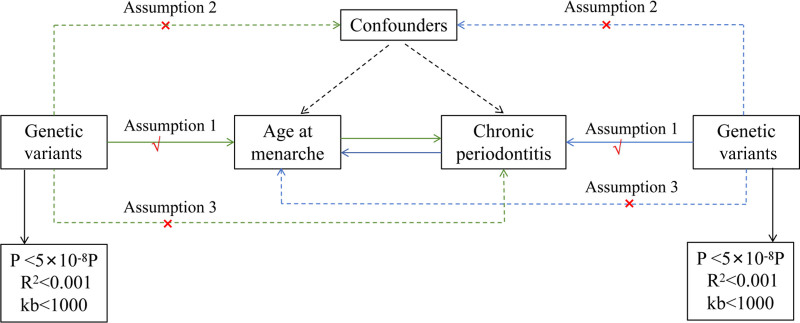
Overview of the Mendelian randomization (MR) study. Assumption 1: genetic variants are robustly associated with exposure. Assumption 2: genetic variants are not associated with confounders. Assumption 3: genetic variants affect the outcome only through the exposure of interest.

We obtained data on AAM and periodontitis from the IEU public database (https://gwas.mrcieu.ac.uk/). For age of menarche, we used genetic variants as IVs, which were identified through genome-wide association studies with stringent significance thresholds (*P* < 5 × 10^−8^), *r*^2^ = 0.001, kb = 10,000. For single nucleotide polymorphism (SNP) selection, we included IVs with an *F*-statistic >10 to ensure their validity in the MR analysis, where *F* = beta^2^/se^2^. The PhenoScanner database (https://github.com/phenoscanner/phenoscanner) was used to query all selected SNPs for associations with potential confounders. SNPs strongly associated with known confounders were excluded from the analysis. Detailed information on the SNPs can be found in Table S1, Supplemental Digital Content, https://links.lww.com/MD/P609. This study satisfies 3 key conditions for MR: (1) IVs must be strongly associated with exposure, (2) IVs must not be associated with any confounders, and (3) IVs must affect the outcome only through exposure and not through other pathways. The flow of this study is schematized in Figure 1. The chronic periodontitis data used in this study were sourced from a Finnish database, comprising 3046 patients and 195,395 controls. The AAM data were obtained from the UK Biobank database, involving 243,944 individuals. All study participants were of European descent, in order to minimize confounding effects related to population structure. No additional ethical approvals or consents were required for our study as our data were obtained from publicly available online databases and ethical approval and informed consent were obtained for the original study.

### 2.2. Statistical analysis

We performed 2-sample MR analyses to estimate causal associations between AAM and the risk of periodontitis and gingivitis. The primary analysis employed the inverse-variance weighting (IVW) method, a widely used and robust approach that combines SNP-level estimates to generate a causal estimate. To assess the validity of our results and detect potential sources of bias or pleiotropy, we conducted a sensitivity analysis using several methods, including Cochran *Q* statistic, MR-PRESSO, MR-Egger regression, and the leave-one-out test for pleiotropy. Additionally, we used alternative MR methods, such as MR-Egger, weighted median, simple mode, and weighted mode, to evaluate the consistency of estimates and assess the sensitivity of our results to potential violations of MR assumptions. By detecting outliers or directional pleiotropy, we aimed to bolster the reliability of our causal inferences. All analyses were carried out in R software 4.3.1 utilizing the “Two Sample MR.”

## 3. Results

### 3.1. Causal effects of AAM on chronic periodontitis

**Figure 2.**
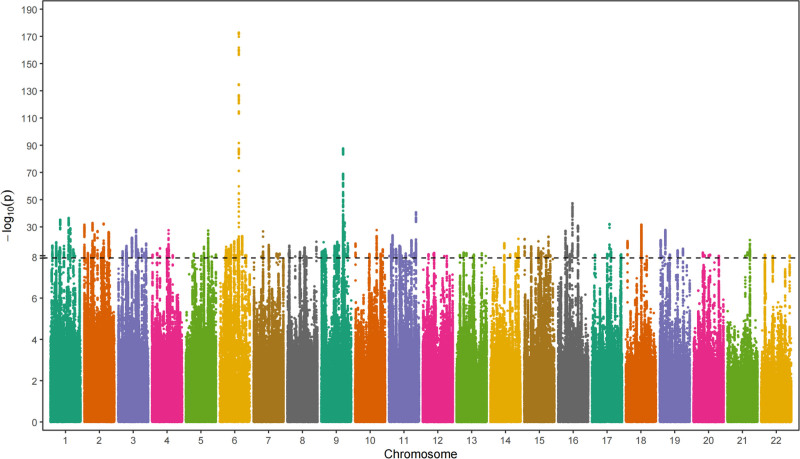
Manhattan plot of SNPs significantly associated with age at menarche. The association between single nucleotide polymorphisms (SNPs) and age at menarche. The *x*-axis represents the chromosomal position of each SNP, arranged by chromosome, with equal spacing across all chromosomes. The *y*-axis shows the negative logarithm of the *P*-value (−log_10_), indicating the strength of association. The dotted line denotes the genome-wide significance threshold (*P* = 5 × 10^−8^).

**Figure 3.**
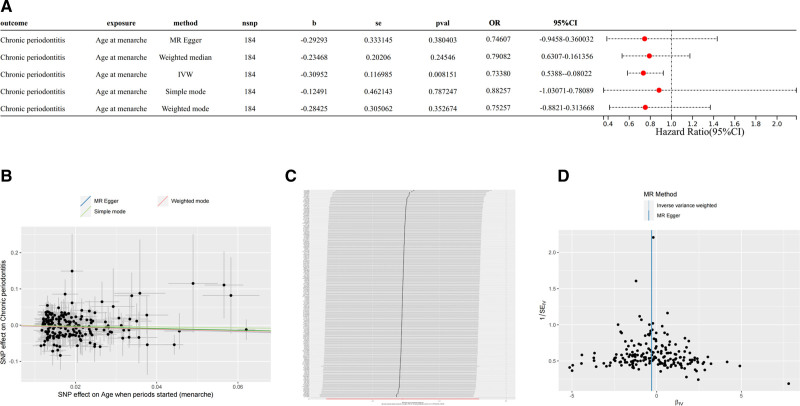
Mendelian randomization analysis of the causal effect of age at menarche on chronic periodontitis. (A) Forest plot displaying the causal estimates derived from 5 Mendelian randomization (MR) methods, including inverse variance weighted (IVW), MR-Egger, weighted median, simple mode, and weighted mode. The IVW method showed a statistically significant inverse association, indicating that an earlier age at menarche may reduce the risk of chronic periodontitis. (B) Scatter plot showing SNP-specific associations between age at menarche and chronic periodontitis, with regression lines for different MR methods. (C) Leave-one-out sensitivity analysis illustrating the robustness of the IVW causal estimate; each line represents the causal estimate after excluding one SNP at a time. (D) Funnel plot assessing the presence of directional pleiotropy. SNP = single nucleotide polymorphism.

Based on the above IV screening conditions, 192 SNPs were identified as IVs for AAW in this study. The Manhattan distribution of the SNPs is shown in Figure 2, and detailed information of the SNPs is shown in Table S1, Supplemental Digital Content, https://links.lww.com/MD/P609. IVW analysis showed a statistically significant correlation between the AAM and a reduced incidence of chronic periodontitis (odds ratio: 0.73; 95% confidence interval: 0.58–0.92; *P* = .0081) (Fig. 3A and B). In addition leave-one-out showed that no single SNP significantly affected the robustness of our results and the funnel plot showed no significant bias (Fig. 3C and D).

### 3.2. Inverse MR analysis

**Figure 4.**
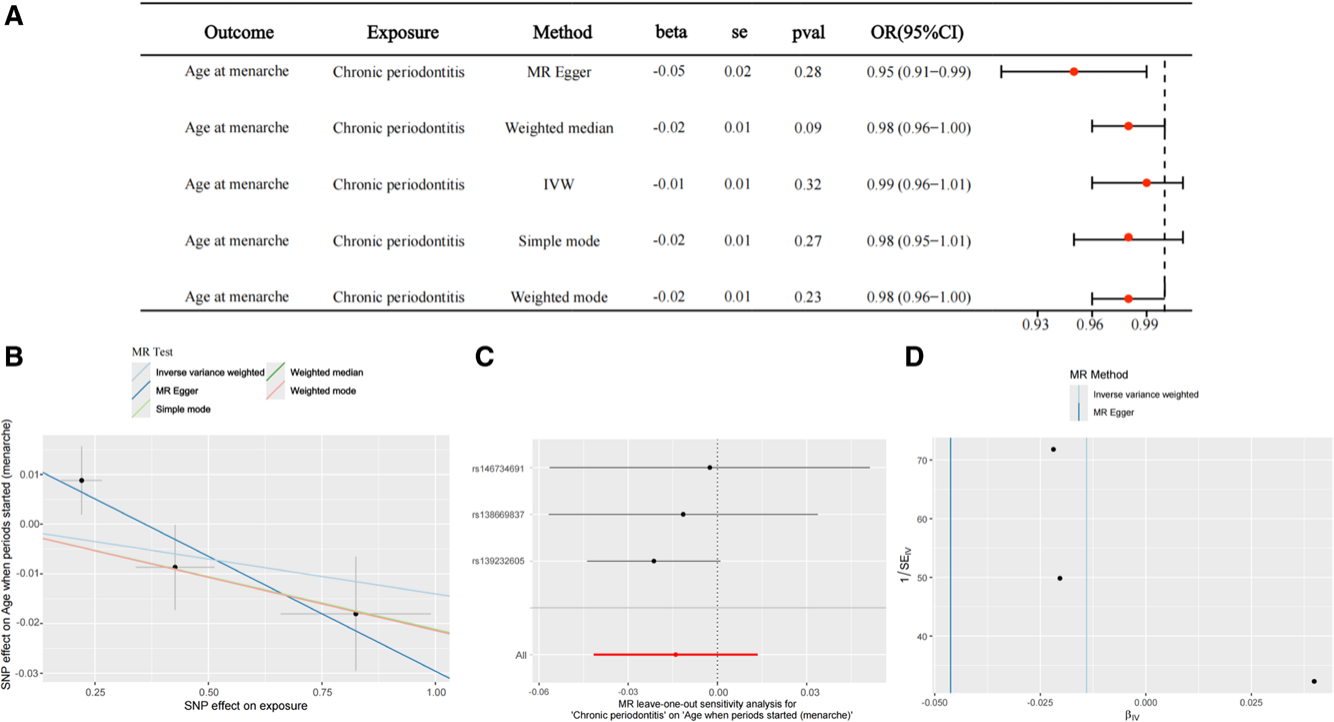
Reverse Mendelian randomization analysis of the causal effect of chronic periodontitis on age at menarche. (A) Forest plot showing causal estimates derived from 5 MR methods: inverse variance weighted (IVW), MR-Egger, weighted median, simple mode, and weighted mode. None of the methods indicated a statistically significant effect, suggesting no strong evidence for a causal influence of chronic periodontitis on the timing of menarche. (B) Scatter plot displaying SNP-specific associations, with regression lines corresponding to different MR methods. (C) Leave-one-out analysis showing robustness of the overall causal estimate; each line represents the result when one SNP is excluded from the analysis. (D) Funnel plot assessing potential directional pleiotropy. MR = Mendelian randomization, SNP = single nucleotide polymorphism.

Using chronic periodontitis as the exposure and AAM as the outcome, no causal association was found by MR analysis (odds ratio: 0.99; 95% confidence interval: 0.96–1.01; *P* = .32) (Fig. 4).

### 3.3. Sensitivity analysis

In sensitivity analyses, SNP heterogeneity was first determined using Cochran *Q* test in IVW and MR-Egger analyses (IVW: *Q* = 152.55, *P* = .95; MR-Egger: *Q* = 152.55, *P* = .94) (Table 1) using the MR-Egger Intercept test in order to assess the potential for directed pleiotropy (MR-Egger Intercept = −0.0003, *P* = .95). Reverse MR analysis confirmed the absence of reverse causal associations, and the above results indicate that our results are robust. The sensitivity analysis of reverse MR is shown in Table 2.

**Table 1:** Sensitivity analyses for the causal effect of age at menarche on chronic periodontitis.

Exposure	Outcome	Heterogeneity			MR‐Egger regression	
		Method	*Q*	*Q*-*P* value	Intercept	*P* intercept
Age when periods started (menarche)	Chronic periodontitis	MR-Egger	152.55	.94	−0.00030	.95
		IVW	152.55	.95		

IVW = inverse-variance weighted, MR = Mendelian randomization.

**Table 2:** Sensitivity analyses for the causal effect of chronic periodontitis on age at menarche.

Exposure	Outcome	Heterogeneity			MR‐Egger regression	
		Method	*Q*	*Q*-*P* value	Intercept	*P* intercept
Chronic periodontitis	Age when periods started (menarche)	MR-Egger	0.63	.42	0.009	.34
		IVW	3.44	.17		

IVW = inverse-variance weighted, MR = Mendelian randomization.

## 4. Discussion

Insights into the impact of sex hormones on oral health are best derived from the association between the AAM and the incidence of developing periodontitis. As previous studies demonstrate, there is a common ongoing inflammation associated with periodontitis, which afflicts the periodontal tissues and if not treated, can lead to loss of teeth.^[10]^ It is necessary to understand periodontitis risk factors for the development of appropriate, effective, and targeted therapeutic strategies. In this study, MR analysis using powerful genetic IVs offered a useful tool to facilitate the study of causal relationships and mechanisms of the association.

In women, estrogen is the main sex hormone and is critical in regulating different physical processes such as the immune system and bone metabolism.^[11]^ Different life stages such as during puberty, menstrual cycle, and menopause are times when there are changes in the estrogen levels which are crucial in the regulation of immune system. Estrogen levels are highest during puberty and coincides with menarche which is the first menstruation cycle.^[12]^ During these stages, estrogen significantly impacts both the acquired and congenital immune systems by modulating immune cell proliferation, functionality, and cytokine secretion. Adolescents with raised estrogen levels have been shown to demonstrate increased multiplication and functioning of immune cells such as macrophages, dendritic cells, and T cells, and increased secretion of anti-inflammatory cytokines making it easier for the control of over active immune responses.^[13]^ The regulation of the immune response in adolescents is important in the development of the immune system and for homeostatic balance of the tissues. In addition, estrogen also adjusts the production of inflammatory mediators and immune factors in periodontal tissues and therefore can be instrumental in the pathogenesis of periodontal diseases. Estrogen exactly regulates the process of inflammation in periodontal tissues via the regulation of pro-inflammatory cytokines and mediators’ expression, which can exert some influence on the initiation or exacerbation of periodontitis.^[14]^

Bone metabolism affected by estrogen is well-studied already. Estrogen is known to stimulate bone formation and at the same time suppress bone resorption.^[15]^ In the case of periodontitis which is coupled with alveolar bone loss, it is possible that estrogen bone protective effects help retain periodontal tissue integrity and alleviate periodontitis severity.^[16]^ The association between periodontal health and sex hormone is bidirectional; periodontitis may cause systemic inflammation which could lead to hormonal dysfunction and affect the menstrual cycle. Hormones and periodontal disease are likely interrelated but additional studies are required to delineate its bidirectional nature. The occurrence and severity of periodontitis is affected greatly by gender. Some hormonal changes increase the women’s susceptibility to periodontal diseases at certain stages in their life. Studies suggest that women during pregnancy and around menopause are more likely to have periodontal disease due to considerable fluctuation of hormonal levels. During pregnancy, increased levels of estrogen and progesterone tend to amplify the periodontal tissue inflammation, resulting in pregnancy-associated gingivitis and periodontitis in some cases. The perimenopausal stage, characterized by reductions in estrogen, is also associated with increased frequency of periodontal inflammation. The lowered estrogen levels could impair immune system functionality and bone metabolism, leading to more severe periodontal diseases.^[17,18]^

Oral contraceptives as well as estrogen replacement therapy are also considered in the context of their effects on periodontal health. These therapies, termed as hormonal modulation therapies, may alleviate negative impacts of hormonal fluctuations on periodontal tissues, thus possibly improving oral health. Earlier investigations show that estrogen replacement therapy has a protective effect on bone metabolism which might decrease the likelihood of bone loss due to periodontal disease as well as the development of periodontitis in postmenopausal women.^[19,20]^ However, caution should be used when considering the relevance of hormonal therapies as some reports indicate that such therapies are capable of altering inflammatory markers and therefore, having baffling effects on periodontal health.

Given the critical role of hormones in periodontal diseases, hormone level regulation may be an adjunctive strategy in the prevention or treatment of periodontitis, particularly in women undergoing significant hormonal changes such as pregnancy, menopause, and hormone therapy. However, more studies are needed to explore the long-term effects of hormonal regulation and treatment on periodontal health, as well as to determine the most appropriate hormonal therapies for prevention.

Although our MR analysis provided compelling evidence for the association of the AAM with periodontitis, there are major limitations of our study. MR analysis relies on certain assumptions: the IVs are valid, and there is absence of horizontal pleiotropy. While sensitivity analyses were conducted to determine the robustness of the results and to reduce potential biases, the effects of unmeasured pleiotropy or confounders may still affect the reported associations.^[21]^ Moreover, this study was based on data from the European population; therefore, caution needs to be exercised while generalizing the result to other ethnic groups. Improving the external validity of our results will be the necessity of conducting the study in diverse populations. There is absolutely no doubt that periodontitis is a multifactorial disease, and many exogenous factors play a crucial role in its development and progression. Such findings deserve sharing to expand the knowledge of the genetic basis for this common disease, which has been comparatively underexplored to date.

Furthermore, considering the rising prevalence of periodontal diseases worldwide, our findings have implications for public health and clinical practice. Early AAM has been associated with various health risks, including metabolic disorders, cardiovascular diseases, and breast cancer.^[22,23]^ Overall, these studies have suggested that women with earlier AAM tend to suffer from increased incidence of developing these diseases, possibly due to prolonged estrogen exposure and its effects on the metabolism and cardiovascular systems. However, factors like socioeconomic status, certain lifestyle decisions, and other health issues which have bearing on the age of menarche as well as the occurrence of diseases all combine to make these findings complex.

Our MR analysis reduced the impact of confounding variables through the use of genetic variations as proxy variables for AAM. MR analysis examined genetic variables of menarche timing to achieve stronger and less biased estimation of causal relationship between early AAM and periodontitis. Our MR analysis supported prior studies that early AAM promoted periodontal disease outcomes. However, earlier observational studies showed differences that might result from uncontrollable confounding variables in conventional epidemiological research. Magnetic resonance based on genetic variables, on the other hand, is less vulnerable to these confounding variables and can therefore offer more convincing proof of the causal relationship.

This study identified a negative correlation between AAM and periodontitis which expands the range of adolescent timing health outcomes to encompass oral health. Our results suggested that an early AAM may be associated with periodontitis regardless of other confounding variables. Healthcare professionals ought to recognize this risk and begin to incorporate oral health assessments and prophylactic measures into the management of patients who have an early menarche.

## 5. Conclusions

In summary, the MR analysis provided strong evidence supporting the association between AAM and reduced periodontitis risk.

## Author contributions

**Data curation:** Yan Hou, Weilun Cai.

**Supervision:** Jiandong Ban.

**Validation:** Hua Xu.

**Writing – original draft:** Jiandong Ban.

## Supplementary Material


